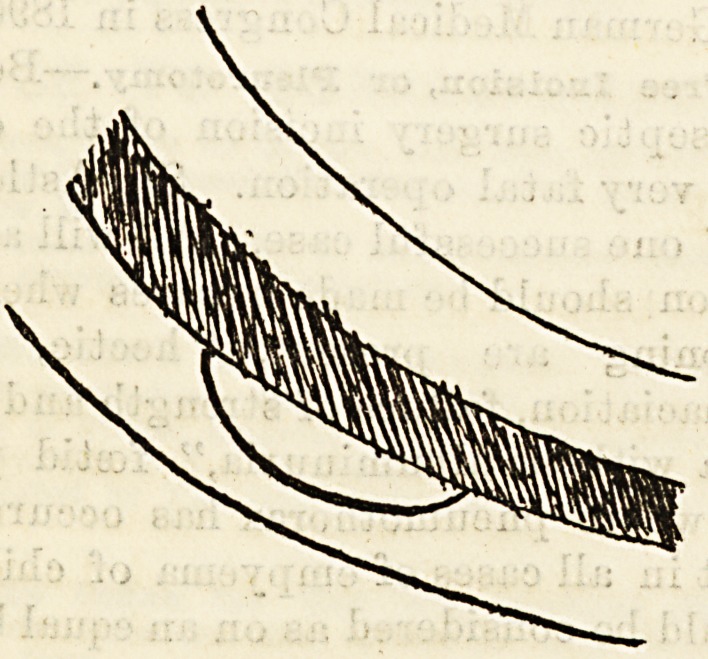# Notes on the Methods of Performing Paracentesis of the Various Cavitives

**Published:** 1894-10-06

**Authors:** John Poland

**Affiliations:** Surgeon to the Miller Hospital, Greenwich


### MEDICAL PROGRESS AND HOSPITAL CLINICS.

[*The Editor will be glad to receive offers of co-operation and contributions from members of the profession. All letters should be addressed to* THE EDITOR, THE LODGE, PORCHESTER SQUARE, LONDON, W.]

### NOTES ON THE METHODS OF PERFORMING PARACENTESIS OF THE VARIOUS CAVITIES.

By JOHN POLAND, F.R.C.S., Surgeon to the Miller Hospital, Greenwich.

**Empyema.**—As a broad rule, purulent collections in the chest demand more active measures than serous collections. Indeed, we may assert that empyema is essentially a surgical affection; its conditions are so very different from serous effusions. We have the choice of three methods of treatment. (1) Repeated aspirations. (2) Introduction of drainage tube—subaqueous syphonage. (3) Pleurotomy, or free incision.

**First, as to repeated aspirations**, the case of the adult differs from that of the child. In the former aspiration should hardly ever be employed, the delay in making a free incision may lead to death by hectic, perforation of bronchus, or internal abscess. Absorption cannot be relied upon in adults; whereas in the case of children the treatment of empyemata by repeated aspiration or the repeated use of the trocar and cannula has occasionally proved successful. After one or two tappings there is sometimes found to be no further secretion of pus. These cases are, however, exceptional, and must only be regarded in this light. On the other hand, delay in many cases has proved disastrous to the lung.

The whole of the fluid, or as much as possible, should be removed. Towards the end of the withdrawal of the fluid, the cannula should be directed downwards and backwards to avoid the lung blocking the end of the tube. Injections should never be made; if hectic is set up after three or four tappings, then incise. Twenty or even more aspirations in empyema in children belong to bygone surgery, and should never be attempted. Aspiration for empyema in children should never be persisted in unless there is a distinct tendency to absorption. If a cure does not follow after two, or at the most three, aspirations, then the collections should be treated like any other abscess and incised. Some go so far as to declare that a spontaneous cure can never be properly relied upon, and that even after absorption following aspiration it is a question "whether the caseous mass left behind may not set up tubercular disease, or the slow absorption leave a puny and weakly child, or the lung contract adhesions which may prevent its full expansion, which would not have been the case had a free incision been made."

**Secondly. Continuous Subaqueous Drainage** is still less satisfactory than in serous effusions. After the puncture, by means of the trocar or cannula, the tube at the end of the cannula is passed into an antiseptic solution below the bed (a cannula with double tube for washing out is sometimes employed), or a drainage tube may be introduced into the chest through a cannula between the ribs. On the cannula being withdrawn the tube is held by the soft parts. Unfortunately, these tubes or cannulas often get loose, and air getting in the fluid may become septic, involving a serious risk to the patient. These methods are seldom used, and have little to recommend them, although they received strong support at the German Medical Congress in 1890.

**Thirdly. Free Incision, or Pleurotomy.**—Before the days of antiseptic surgery incision of the chest was considered a very fatal operation. Sir Astley Cooper only knew of one successful case. All will admit that a free incision should be made in cases when signs of septic poisoning are present, "hectic, sweating, diarrhœa, emaciation, failure of strength and appetite, or anarsarca without albuminuria," fœtid pus after tappings, or where pneumothorax has occurred. But I believe that in all cases of empyema of children free incision should be considered as on an equal basis with thoracentesis. The proportion of cures by simple aspiration and by free incision has not been determined because—"(1) Unsuccessful cases of both are not usually recorded. (2) Failure of treatment by aspiration and later treated by incision. (3) Incision often delayed beyond what the strength of the patient will permit." Incision is one of the most successful operations of modern surgery, and the absolute cure of empyema is now accomplished with the greatest certainty.

The operation is shortly as follows, and is carried out with the strictest attention to antiseptic and aseptic details. The patient being put under chloroform, not ether, an incision two inches long is made in one of the lower intercostal spaces in the same site in the axilla as that for paracentesis, taking care to avoid the long thoracic veins, or in the eighth or ninth interspace behind, just external to, or below the angle of the scapula. The knife should not be plunged into the cavity, but a careful dissection should be made down to the pleura along the upper border of the rib. A grooved director should then be passed through the pleura, which should be divided upon it. The finger or fingers may be introduced into the cavity to remove any masses of lymph. The chloroform should now be stopped, and a large-sized drainage tube introduced within the pleural cavity. The ingress of air must be prevented as much as possible, and the exit of pus allowed, by a good layer of carbolic or eucalyptus gauze or other volatile antiseptic dressing. This should be carefully packed round the tube with a layer of jaconet towards the exterior. The dressings must be changed every twenty-four hours, or whenever soiled. The lung will by these means be permitted to expand slowly and effectually.

The pleural cavity should not be washed out after the pus is evacuated; it is a dangerous procedure, and often keeps up the discharge. Injections are only required when there is great fœtor.

The sooner the pus is let out by free incision the more rapid, the more complete, the more certain the recovery, with a diminution in the risk of the lung becoming inexpansible.

To postpone free incision means very often a resulting inexpansible lung, a very grave condition. We must, therefore, always interfere in recent cases, and not allow them to become old. If the wound is kept sweet with Listerian and other precautions there is no more danger than in aspiration, and, as already stated, the same applies to long-standing cases of serous effusion. We get the happiest results from this form of treatment.

Under this heading I might almost place the additional removal of a slice of the width of the rib in the case of children, such as I have performed for some years with equal success in double as well as single empyema. After the periosteum has been incised longitudinally and separated, two-thirds or more of the width of the bone is removed from the upper part of the lower rib by means of a chisel or stout knife. The portion removed should be of an elliptical shape, as in the diagram, and the pleura opened in the usual way. In the large proportion of cases this gives plenty of room for exploration and insertion of the tube. By the time new bone is produced from the periosteum—and this is mostly below the tube—the latter can, as a rule, be removed. The outline of the rib is in this short space of time so perfect that the appearance is the same as if only a simple pleurotomy had been done. In fact, it does not render the operation of simple incision any more complicated, and I have never had necrosis occur as the result of this procedure. In this connection it must be emphasized that resection of portions of the whole width of the ribs is only required in exceptional cases in children, where drainage is insufficient, or the simple incision has failed from the insufficiency of the filling in of the chest, or the ribs being so close together that the removal of a slice of the width of the bone does not allow enough room.

Always explore with the needle before aspiration or pleurotomy. Pus cannot be positively diagnosed, although it may very often be shrewdly suspected.

**Paracentesis of the Pericardium** for purulent collections of rheumatic, septicæmic, or pneumonic origin, or due to infective osteomyelitis, scarlatina, or other of the eruptive fevers, has now been successfully performed in many cases (especially rheumatic ones). (1) By trocar and cannula. (2) By aspiration, guided by the rules already mentioned for the chest; a small amount of fluid only is drawn off; the site usually chosen has been the fourth or fifth interspace close to the sternum, the trocar being directed upwards and outwards. (3) By incision under antiseptic precautions and insertion of a drainage tube. This has been successfully performed a few times. It may be undertaken in excessive distension of the cavity, seriously embarrassing the action of the heart, or in cases where the effusion refuses to be absorbed by ordinary treatment, or for purulent pericarditis after failure of aspiration. There is no reason why a collection of pus in the pericardium should not be evacuated like any other abscess.

**Tapping the Abdomen.**—All will agree that this should be performed in all cases where the collection of fluid in the peritoneal cavity interferes with the action of the diaphragm, where there are signs of pressure upon the abdominal or thoracic viscera, as indicated by the suffering and distress of the patient. There are four modes of removing the fluid:—

1. By Southey's capillary tubes, which are left in position for a long time; they are said to cause less collapse and no risk of peritonitis.

2. By aspiration, usually in the middle line between pubis and umbilicus, but the puncture may be made wherever there is dulness on percussion, so as to avoid wounding the intestine, which might happen when the intestine is adherent to the wall, or is situate in front of the fluid swelling. The aspiration is only preferable to the trocar and cannula when the fluid is localised, or where there is any doubt as to diagnosis.

3. Permanent drainage by means of a rubber tube has been recommended, but offers no advantage except in hopeless cases. The results are far less favourable than by repeated tapping.

4. The best method is by trocar and cannula, with lateral branch and syphon attached. The end of the tube is placed in a basin of antiseptic fluid (below the bed), which may be raised or lowered according to the pressure required. The operation is performed with the patient lying on the side, or sitting in a chair if not in a very weak condition. The instruments should be perfectly aseptic, and the skin of the patient and the operator's hands perfectly cleansed. The bladder is first emptied, and the trocar plunged (not too deeply) either in the middle line or elsewhere, taking care to avoid the deep epigastric vessels. If the flow from the tube stops, it is probably due to omentum, which may be displaced by a blunt-pointed probe passed through the cannula. The usual bandage round the abdomen should be gradually tightened to avoid collapse, and then fastened on without being relaxed. Collodion must be applied to the puncture opening.

Tapping, even when frequently performed, is absolutely devoid of risk in these days of aseptic and antiseptic surgery. Tap early, but do not retap too soon, even if the cavity fills up again, say after two or three weeks. In previous years the operation has been delayed as long as possible. Nearly all text-books recommended paracentesis when other measures have failed—purgatives, tonics, and diuretics. This is what Niemeyer says: "The abdomen should only be tapped where life is immediately endangered by obstruction of the respiration, or by a threatened gangrene of the skin." But now we say the operation should be performed early, not only whenever there is any pain or discomfort from the ascites, but that it should be adopted as a routine method of treatment, combined with drugs, &c. It improves the condition of the patient, and assists the cure of the disease. It should not be postponed till urgent symptoms arise and the abdominal distension so extreme as to impair the abdominal organs. Ascites should not be looked upon as the beginning of the end. A large number of cases of apparent permanent recovery are now recorded where tapping has been adopted as a curative method of treatment. Many cases are known to me that have died of other diseases several years after repeated tappings—sometimes twenty or thirty times. A marked case occurred twelve years ago in the practice of Mr. Wm. F. Hearnden of Sutton. A man between forty and fifty years of age was tapped by him nine or ten times, and fifteen or twenty quarts of fluid drawn off on each occasion. He is now alive and quite recovered. Micelli recorded a case of ascites in a young woman, where paracentesis was performed thirty-four times. After the last there was no reaccumulation, and the patient afterwards enjoyed good health.

## Figures and Tables

**Figure f1:**